# Skeletal Muscle Mass Index and Body Fat Percentage Reflect Different Nutritional Markers Independent of BMI in Underweight Women

**DOI:** 10.3390/nu17111766

**Published:** 2025-05-23

**Authors:** Eri Hiraiwa, Risako Yamamoto-Wada, Kanako Deguchi, Chihiro Ushiroda, Hiroyuki Naruse, Katsumi Iizuka

**Affiliations:** 1 Department of Clinical Nutrition, Fujita Health University, Toyoake 470-1192, Japan; 51022099@fujita-hu.ac.jp (E.H.); risako.wada@fujita-hu.ac.jp (R.Y.-W.); kanasakuran@gmail.com (K.D.); chihiro.ushiroda@fujita-hu.ac.jp (C.U.); 2 Faculty of Medicine, Fujita Health University, Toyoake 470-1192, Japan; 3 Health Management Center, Fujita Health University, Toyoake 470-1192, Japan; hnaruse@fujita-hu.ac.jp; 4 Food and Nutrition Service Department, Fujita Health University Hospital, Toyoake 470-1192, Japan

**Keywords:** skeletal muscle mass index, SMI, body fat percentage, BF%, BMI, body mass index

## Abstract

**Background/Aim:** Skeletal muscle mass index (SMI) and body fat percentage (BF%) are components of body mass index (BMI) but are considered to play independent roles. We aimed to clarify whether SMI and BF% are associated with nutritional markers independent of BMI in underweight women. **Methods:** This retrospective observational study included a total of 102 women aged 20–65 years who were referred to the outpatient nutrition evaluation clinic from 2022 to 2024 with a body mass index (BMI) < 17.5. We performed a multivariate analysis with SMI and BF% as independent variables and BMI, BMI ratio (present-to-age 20 ratio), grip strength, and biochemical nutritional indicators (vitamin B_1_ level (ng/mL), cholesterol level (mg/dL), lymphocyte count (/μL), and HbA1c (%) level) as dependent variables, adjusting for age. **Results:** Women aged 30.9 ± 10.2 years (yo) with a BMI of 17.0 ± 0.7 participated in this study. BMI (kg/m^2^) was positively associated with SMI (kg/m^2^) (β (95% CI): 1.6 [1.4, 1.9], *p* < 0.001) and BF% (0.2 [0.1, 0.2], *p* < 0.001), and the BMI ratio (present-to-age 20 ratio) was positively associated only with BF% (0.5 [0.05, 0.9], *p* = 0.03). Grip strength was positively associated with SMI (4.0 [1.4, 6.6], *p* = 0.003), and lymphocyte count was positively associated with BF% (β (36.2 [6.0, 66.5], *p* = 0.02). BMI was not associated with grip strength or lymphocyte count. Vitamin B_1_, cholesterol, and HbA1c were not associated with SMI, BF%, or BMI. **Conclusions:** These results indicate that SMI reflects BMI and grip strength, whereas BF% reflects BMI, the BMI ratio (present to age 20), and lymphocyte count. In addition to BMI and SMI, changes in BF% should also be noted in underweight women.

## 1. Introduction

Among women in Japan, underweight is a particularly serious problem [1,2]. According to the 2022 National Health Survey of Japan, 4.3% of men are underweight, whereas 11% of women are underweight [2]. In particular, 19.1% of women in their 20s are underweight [2]. This trend has not changed over the past 10 years [2]. At Fujita Health University, 14% of all women, 17% of those aged 20–29 years, 14% of those aged 30–39 years, 9% of those aged 40–49 years, and 8% of those aged 50–65 years are underweight [3]. Health literacy represents the personal knowledge and competencies that accumulate through daily activities, social interactions, and across generations. The general population usually knows less about health than experts do; however, underweight is considered a socioenvironmental problem since it is seen even in medically literate populations and improves with age. Health problems associated with underweight include osteoporosis, menstrual abnormalities, infertility, anemia, glucose intolerance, and, as we have reported, various vitamin deficiencies [4,5,6,7,8]. The prevalence of vitamin D deficiency is low among underweight women, whereas folic acid and vitamin B_12_ deficiencies are observed in approximately 20% of underweight women, along with vitamin B_1_ deficiency, which is rarely observed today [4]. Thus, underweight is a condition that increases the risk of disease due to malnutrition, not just weight loss [1,2].

Undernutrition, especially a loss of lean body mass, leads to reduced physical function and increases the risk of frailty and sarcopenia. Lean body mass (LBM) is a part of body composition that is defined as the difference between total body weight and body fat weight. Undernutrition is widely considered very important for maintaining lean body mass (muscle mass). Skeletal muscle mass index (SMI) is known to be a good indicator of muscle mass [9,10,11]. Grip strength is known to be related to life span [12], and the strong association between SMI and grip strength is also well documented [13,14]. On the other hand, adipose tissue is particularly related to menstruation, and a low body fat percentage (BF%) may also be problematic [15,16,17]. A minimum ratio of fat to lean mass is normally necessary for menarche (approximately 17% fat/body weight) and the maintenance of female reproductive ability (approximately 22% fat/body weight) [17]. Among elderly individuals, the risk of a low muscle percentage and high BF%, as observed in those with sarcopenic obesity, has also been discussed [18,19]. There is concern that such conditions may be associated with diseases such as glucose intolerance and dyslipidemia, in addition to muscle weakness. However, the current status of body size (SMI and BF%) in underweight women in Japan is unknown.

The controlling nutritional status (CONUT) score has long been known as an index used to screen nutritional status. The subjective global assessment (SGA) and full nutritional assessment (FNA) scores are correlated with the degree of undernutrition according to the CONUT score [20]. A higher CONUT score is associated with an increased risk of death and complications in the hospital, longer hospital stays, increased risk of sepsis, increased risk of in-hospital mortality in patients with ischemic stroke, and increased risk of in-hospital mortality in elderly patients [21,22,23]. It is unclear why the CONUT score is based on the cholesterol level, albumin level, and lymphocyte count; however, lymphocyte count is used as a measure of immune status, the albumin level is used as a measure of protein synthesis, and the cholesterol level is used as a measure of lipids. It is poorly understood whether nutritional parameters such as lymphocyte count and cholesterol level are related to body size in individuals with poor nutritional status.

There are still no clear threshold SMI or BF% criteria for defining poor nutritional status [24,25,26,27,28], and although lower SMI thresholds have been observed for men and women according to the diagnostic criteria for sarcopenia, the thresholds for elderly people are not met, and no criteria for young and middle-aged people have been identified [25]. The body fat percentage of healthy individuals has been reported to vary with age, body size, and race [28]. It has long been noted that visceral fat accumulation is important in the pathogenesis of metabolic syndrome, and the abdominal circumference and visceral fat area are sometimes measured [29,30]. However, in recent years, it has become possible to measure body composition easily, and there is an opinion that it is better to evaluate obesity or overweight according to body fat percentage rather than BMI [31]. Under these circumstances, we believe that it is necessary to examine how SMI and BF% affect nutritional indices.

Therefore, the purpose of this study was to clarify whether SMI and BF% reflect nutritional markers distinctly from BMI and to clarify the significance of SMI and BF% measurements in underweight women. First, body size and nutritional indices according to age, SMI, size, and BF% were determined. Next, multivariate analysis was used to determine whether various nutritional indices were associated with SMI and BF% when adjusted for age. In the present study, BMI was positively associated with SMI and BF%. In addition, higher skeletal muscle mass was associated with a lower BF%. The multivariate analysis revealed that SMI was positively associated with current BMI and grip strength, whereas BF% was positively associated with BMI, BMI ratio (present-to-age 20 ratio), and lymphocyte count. Since grip strength and lymphocyte count were not found to be associated with BMI, it is clinically meaningful to measure SMI and BF% in addition to BMI. Currently, reference values are not always clear; therefore, these values are often determined by looking at changes in values over time within individuals. Given that the significance of SMI and BF% in underweight individuals has been clarified, SMI and BF% should be used independently of BMI.

## 2. Materials and Methods

### 2.1. Subjects

One hundred and two female patients aged 20–59 years participated in this study. The criteria for this study were as follows: (1) aged 20–59 years and (2) referred to the outpatient nutrition evaluation (clinical nutrition) clinic due to a BMI of <17.5. Among those who had undergone the Fujita Health University staff health examination from 2022 to 2024 were eligible for this retrospective, cross-sectional observational study. The patients were referred to the Clinical Nutrition Department for nutritional evaluation. Patients who were already taking vitamin supplements or drugs at the time of the initial visit were excluded. Pregnant women and patients being seen in the psychiatry department for the treatment of anorexia nervosa were also excluded because they were seen by their primary care physician. During the outpatient consultation, the attending physician checked for signs of weight loss (fever, intentional weight loss, appetite, and edema) via an interview and physical examination, but none of the patients complained of any obvious findings. Most underweight patients, with the exception of those with low body weights, did not show notable findings on physical examination. To ensure that research subjects had the opportunity to opt out, the principal investigator included the following information on the website of the Department of Clinical Nutrition, Fujita Medical School (the approval date: from 27 December 2024 to 31 March 2027). The study was conducted according to the principles of the Declaration of Helsinki and was approved by the Research Ethics Committee of Fujita Health University (approval number HM24-333 (approval date: 27 December 2024)).

### 2.2. Data Collection

For the 102 female patients who visited our department for secondary screening, information on age, sex, BMI, BMI at age 20, BMI ratio (present-to-age 20), grip strength, BF%, SMI, and blood test parameters (glycated hemoglobin (HbA1c), hemoglobin (Hb), prealbumin, albumin, cholesterol, lymphocyte count, vitamin B_1,_ vitamin B_12_, folic acid, and 25-hydroxyvitamin D (25(OH)D)) was obtained from medical records. Body weight (BW), BF%, and skeletal muscle mass were measured with an InBody Dial H20N weight analyzer (Inbody Japan Inc., Tokyo, Japan). Skeletal muscle mass index (SMI) is a measure of skeletal muscle mass weight, which is typically calculated by dividing the appendicular lean mass (muscle mass of the arms and legs) by height squared. BW at age 20 was a self-reported value confirmed during a medical interview at the time of the outpatient visit. The BMI ratio (present-to-age 20) was calculated as the ratio of BW at present to BW at 20 years. In this study, grip strength was calculated as the average strength of both hands. Grip strength was measured with a TOEI LIGHT grip strength tester ST3 T1781 (Toei Light Inc., Souka, Saitama, Japan). Blood tests (hemoglobin) and white blood cell fractions (lymphocyte count) were measured via Sysmex XN-300 (Sysmex, Kobe, Japan). Vitamin B1 was measured by the LC/MS/MS system (Shimadzu corporation, Kyoto, Japan). Vitamin B12 and folate were measured by unicel DxI800 (Beckman Coulter, Tokyo, Japan). 25OHD was measured by Lumiplulse G1200 (Fujirebio, Tokyo, Japan).

Prealbumin, albumin, and plasma lipid concentrations (TC, TG, and HDL-C) were measured via a Hitachi LABOSPECT008 (Hitachi High-Tech Corporation, Tokyo, Japan), and HbA1c was measured via an A1c HA-8190 (Arkray, Kyoto, Japan).

The CONUT score, a screening tool for detecting inadequate nutrition on the basis of a low albumin level, cholesterol level, and lymphocyte count, was calculated [20]. The CONUT score is based on the cholesterol level, albumin level, and lymphocyte count, which are scored on a four-point scale; scores are categorized as normal nutritional status (0–1), mild malnutrition (2–4), moderate malnutrition (5–8), and severe malnutrition (9–12) [20]. Plasma vitamin B_1_ deficiency, vitamin B_12_ deficiency, plasma folate deficiency, vitamin D deficiency, and anemia were defined as plasma vitamin B_1_ levels < 24 ng/mL, plasma vitamin B_12_ levels < 200 pg/mL, plasma folate levels < 4 ng/mL, plasma 25(OH)D levels < 20 ng/mL, and Hb < 11.5, respectively.

### 2.3. Statistical Analysis

Because this was an exploratory study, no sample size was set. The values are presented as the means (standard deviations; SDs).

First, age (20–29, 30–39, and 40–65 years), SMI (quartile range) Q1–Q4, and BF% (quartile range) Q1–Q4 were compared between groups via one-way analysis of variance, dividing patients into age groups or SMI quartile groups. The results are presented as the means (SDs).

Chi-square tests were performed for group comparisons of the presence of vitamin deficiency, the CONUT score, and the presence of anemia.

Next, multivariate analysis was performed with BMI, weight at age 20, and the ratio of current weight to age 20 weight as dependent variables, and SMI, BF%, and age as independent variables. Next, multivariate analyses were conducted with grip strength, vitamin B_1_ level, total cholesterol level, lymphocyte count, and HbA1c level as dependent variables, and SMI, BF%, and age (Model 1), or BMI and age (Model 2) as independent variables. SPSS 29.0.20 (20) (IBM Japan) and GraphPad Prism 10 (GraphPad) were used for statistical analysis. *p* < 0.05 was considered statistically significant.

## 3. Results

### 3.1. Patient Backgrounds Were Classified by Age, SMI, and Body Fat Percentage

#### 3.1.1. BMI, BMI at 20 Years, and BMI Ratio (Present-to-Age 20 Ratio) by Age, SMI, and Body Fat Percentage

The importance of lean body mass, or SMI, in undernutrition, has been emphasized in terms of mortality, but the significance of BF% is less clear. Furthermore, the significance of BMI and BF% measurements for nutritional status remains unclear. In other words, it is unclear whether SMI and BF% reflect nutritional status. In this study, we compared nutritional status across age (20–29, 30–39, and 40–65 years), SMI, and BF% quartile ranges. We subsequently identified factors associated with SMI and BF%. The total sample included 102 participants (20–29 yo (n = 64); 30–39 yo (n = 20); 40–65 yo (n = 18)) (Table 1).

First, regarding the participants’ characteristics, mean (SD) age was 30.9 (10.2) years, BMI was 17.0 (0.7) kg/m^2^, BMI at age 20 was 17.4 (1.4) kg/m^2^, BMI ratio (present-to-age 20) was 98.3 (6.7)%, SMI was 7.1 (0.4) kg/m^2^, BF% was 22.0 (4.0)%, and grip strength was 22.5 (4.8) kg (Table 1).

Next, we examined body size and grip strength, grouped by age (20–29, 30–39, and 40–65 years), SMI (quartile range), and BF% (quartile range) (Table 1). There were no significant differences in age between the SMI quartile groups (Q1–Q4) or BF% quartile groups (Q1–Q4) (age: Q1:28.6 (9.9), Q2:31.0 (9.2), Q3:32.3 (10.6), and Q4:31.8 (11.3), *p* = 0.58 and BF%: Q1:33.0 (10.3), Q2:31.8 (12.7), Q3:28.0 (5.6), and Q4:30.5 (10.7), *p* = 0.36) (Table 1). Furthermore, there were no significant differences in BMI between the age groups (20–29, 30–39, and 40–65 years) (*p* = 0.32) (Table 1). However, there was a significant increase in BMI in the higher SMI groups (Q1: 16.6 (0.8), Q2: 16.8 (0.8), Q3: 17.3 (0.6), and Q4: 17.3 (0.4), *p* < 0.001). Similarly, BMI increased as BF% increased (Q1: 16.7 (0.8), Q2: 17.0 (0.6), Q3: 17.0 (0.8), and Q4: 17.3 (0.6), *p* = 0.04) (Table 1).

There was no significant difference in BMI (kg/m^2^) at the age of 20 years among the age groups (20–29, 30–39, and 40–65 years) or BF% groups (Q1–Q4) (*p* = 0.09 and *p* = 0.50, respectively) (Table 1). However, BMI at the age of 20 years (kg/m^2^) increased significantly as SMI increased (Q1: 16.5 (1.0), Q2: 17.2 (1.1), Q3: 17.7 (0.1), and Q4: 18.1 (7.8), *p* < 0.01) (Table 1). There were no significant differences in the BMI ratio (present-to-age 20 ratio) among the age and SMI groups (*p* = 0.36 and *p* = 0.15, respectively), but a lower BF% was associated with a significantly decreased body weight ratio between ages 20 and present (Q1: 95.3 (7.3), Q2: 97.8 (6.4), Q3: 100.0 (5.6), and Q4: 100.4 (6.5), *p* = 0.032) (Table 1). SMI (kg/m^2^) did not differ between groups in terms of age (*p* = 0.2) but decreased with increasing BF% (Q1: 7.4 (0.4), Q2: 7.2 (0.4), Q3: 7.0 (0.3), and Q4: 6.7 (3.2), *p* < 0.001) (Table 1). The mean (SD) BF% was 22.0 (4.0) and did not differ significantly among the age groups (20–29 yo: 22.6 (4.1); 30–39 yo: 20.8 (2.6); and 40–65 yo; 21.2 (4.5), *p* = 0.13), but BF% decreased with increasing SMI (Q1: 25.2 (4.1), Q2: 22.4 (3.9), Q3: 21.6 (2.6), and Q4: 18.9 (2.3), *p* < 0.001) (Table 1). The grip strength did not significantly differ among the age groups or BF% groups (20–29 yo: 22.3 (5.0); 30–39 yo: 21.4 (4.6); and 40–65 yo: 24.1 (3.7), *p* = 0.2 and Q1:23.9 (4.6); Q2:23.3 (5.0); Q3:21.2 (4.4); and Q4:21.5 (4.7), *p* = 0.12, respectively) (Table 1). However, in the SMI group, there was a significant increase in grip strength (kg) among the quartiles (Q1: 20.0 (3.6), Q2: 22.0 (4.4), Q3: 23.2 (4.1), and Q4: 24.7 (5.6), *p* = 0.03) (Table 1). Thus, there were no differences in body size by age group, but in the SMI group, BMI, BMI at age 20, and grip strength (kg) were greater with greater SMI, and the body fat percentage decreased inversely. In the BF% group, the current body weight and BMI ratio (present-to-age 20 ratio) were greater with greater body fat, and skeletal muscle mass inversely decreased.

#### 3.1.2. Vitamin Levels and Frequency of Vitamin Deficiency by Age, SMI, and Body Fat Percentage

We previously reported a relatively high frequency of vitamin deficiency among underweight women [17]. Therefore, we next investigated whether the frequency of vitamin deficiencies differed by age and body size (Table 2).

For vitamin B_1_, the mean (SD) B_1_ level was 30.1 (6.6) ng/mL. Although there were no significant differences in vitamin B_1_ levels among the age or SMI groups, there were differences in vitamin B_1_ levels (ng/mL) according to BF% groups (Q1: 31.2 (9.2), Q2: 29.2 (4.6), Q3: 27.3 (4.1), and Q4: 32.3 (6.29), *p* = 0.032) (Table 2). Vitamin B_1_ deficiency was found in 8% of the subjects, with a high frequency of 22% in the 40–65-year group, compared with 6% in the 20–29-year group and 0% in the 30–39-year group (*p* = 0.03). For folic acid, the overall frequency of folic acid deficiency was 8.1 ± 4.8%, with no significant differences in folic acid deficiency among the age (20–29 yo: 17%, 30–39 yo: 0%, and 40–65 yo: 6%, *p* = 0.08), SMI (Q1: 12%, Q2: 16%, Q3: 4%, and Q4: 15%, *p* = 0.53), or BF% groups (Q1: 12%, Q2: 12%, Q3: 8%, and Q4: 15%, *p* = 0.9) (Table 2). The overall plasma B_12_ level was 289.4 (131.0) pg/mL, and the overall frequency of plasma B_12_ deficiency was 24%. There were no significant differences among the groups with respect to age, SMI, or BF% (20–29 yo: 27%, 30–39 yo: 20%, and 40–65 yo: 22%, *p* = 0.81; Q1: 23%, Q2: 36%, Q3: 16%, and Q4: 23%, *p* = 0.43; Q1: 27%, Q2: 19%, Q3: 17%, and Q4: 35%, *p* = 0.44). The threshold for 25OH deficiency was 11.2 (5.0) µg/dl, and the overall frequency of vitamin D deficiency was 94%, but there were no significant differences in vitamin D frequency when the age, SMI, or BF% groups were compared (Table 2). Thus, the frequencies of vitamin B_1_ deficiency, folate deficiency, vitamin B_12_ deficiency, and vitamin D deficiency were 8%, 12%, 24%, and 94%, respectively, but only vitamin B_1_ deficiency significantly differed among the age groups. In summary, the frequency of vitamin deficiencies was found to be high among underweight individuals regardless of age, with B_1_ deficiency being particularly common in those aged 40–65 years.

#### 3.1.3. Albumin, Lymphocyte, Cholesterol, CONUT Score, Prealbumin, and Anemia by Age, SMI, and Body Fat Percentage

The total cholesterol level, albumin level, and lymphocyte count are used as markers reflecting nutritional status, and the CONUT score, which is based on the decrease in each parameter, is often used [20]. Total cholesterol levels (mg/dL) increased with age: 20–29 years, 174.8 (26.7); 30–39 years, 166.1 (16.5); and 40–65 years, 209.4 (71.2) (*p* < 0.001) (Table 3). On the other hand, total cholesterol levels (mg/dL) were not significantly different among the SMI and body fat percentage groups (SMI: Q1: 183.9 (27.6), Q2: 169.2 (19.4), Q3:180.6 (26.2), and Q4: 182.8 (66.1), *p* = 0.53, and BF%: Q1: 186.3 (62.3), Q2: 181.2 (33.8), Q3: 170.8 (19.4), and Q4: 177.9 (28.2), *p* = 0.57, respectively). There were no significant differences in the serum albumin levels among the age, SMI, and BF% groups (age: 20–29 yo: 4.5 (0.3); 30–39 yo: 4.5 (0.2); and 40–65 yo: 4.4 (0.3), *p* = 0.40; SMI: Q1: 4.5 (0.2), Q2: 4.5 (0.3), Q3: 4.5 (0.4), and Q4: 4.5 (0.3), *p* = 0.97; and BF%: Q1: 4.5 (0.3), Q2: 4.4 (0.3), Q3: 4.5 (0.3), and Q4: 4.5 (0.3), *p* = 0.35, respectively) (Table 3). Lymphocyte count (/μL) decreased with increasing age: 1859.3 (493.8) in the 20–29-year age group, 1739.0 (503.4) in the 30–39-year age group, and 1429.6 (454.1) in the 40–65-year age group. No significant differences were found between the SMI and BF% groups (SMI: Q1: 1846.1 (530.5), Q2: 1787.7 (485.0), Q3: 1683.6 (567.8), and Q4: 1720.2 (467.8), *p* = 0.68; body fat%: Q1: 1620.2 (385.8), Q2: 1732.1 (522.9), Q3: 1751.3 (320.4), and Q4: 1935.3 (694.0), *p* = 0.16) (Table 3). In addition, for 61% of the subjects, the CONUT score was normal, and 39% had mild undernutrition. Among the age groups (20–29, 30–39, and 40–65 years), 30%, 50%, and 61% had mild undernutrition, respectively. There were no differences among the groups in terms of SMI and BF% (Q1: 27%, Q2: 36%, Q3: 44%, and Q4: 50%, *p* = 0.35; and Q1: 54%, Q2: 38%, Q3: 25%, and Q4: 38%, *p* = 0.22, respectively) (Table 3). Thus, total cholesterol levels increased, lymphocyte counts decreased, and the percentage of patients with mild undernutrition increased with increasing age.

Prealbumin is a rapid turnover protein [26,27], and plasma prealbumin levels indicate the amount of protein intake [28]. The overall prealbumin level was 23.6 ± 4.1 mg/dl, and the prealbumin level was not significantly different among the age, SMI, or BF% groups (age: 23.4 (4.3), 23.7 (3.6), 24.0 (4.0), *p* = 0.84; SMI: Q1:23.3(4.0), Q2: 23.2 (4.4), Q3: 24.5 (4.2), and Q4: 23.3 (3.8), *p* = 0.65; BF%: Q1: 23.2 (4.0), Q2: 24.0 (4.7), Q3: 22.1 (3.9), and Q4: 24.8 (3.4), *p* = 0.12, respectively). The overall HbA1c level was 5.4 ± 0.2%, and the HbA1c level was not significantly different among the age and BF% groups (age: 20–29 yo: 5.4 (0.2), 30–39 yo: 5.5 (0.2), and 40–65 yo: 5.5 (0.2), *p* = 0.51, and BF%: Q1: 5.5 (0.2), Q2: 5.5 (0.2), Q3: 5.5.5 (0.2), and Q4: 5.4 (0.2), p = 0.19, respectively). The HbA1c levels were 5.4 ± 0.3%, 5.4 ± 0.2%, 5.5 ± 0.2%, and 5.5 ± 0.2% in SMI quartiles 1–4, respectively (*p* = 0.049) (Table 3). Thus, prealbumin and HbA1c levels were similar among the age, SMI, and body fat percentage groups.

The Hb level was 13.0 ± 1.0, and 11% of the patients had anemia. There were differences in Hb levels among the age groups (20–29 years, 13.0 ± 0.8; 30–39 years, 12.4 ± 1.5; and 40–65 years, 13.4 ± 0.9 mg/dl; *p* = 0.006). The frequency of anemia was 9% in those aged 20–29 years, 25% in those aged 30–39 years, and 0% in those aged 40–65 years; no significant differences in the frequency of anemia were found in the SMI or BF% quartile groups (SMI: 15%, 12%, 12%, and 4%, *p* = 0.58; BF%: 4%, 19%, 13%, and 8%, *p* = 0.31) (Table 3). Thus, the frequency of anemia was highest in the 30–39-year-old group, but the frequencies of anemia in the SMI group and BF% group were similar.

### 3.2. Multivariate Analysis of the Associations Between Body Size and Nutritional Markers

#### 3.2.1. Body Fat Percentage Rather than SMI Is Associated with the BMI Ratio (Present-to-Age 20 Ratio)

The present study focused on SMI and BF% rather than BMI, as it aims to clarify how SMI and body fat percentage affect nutritional status. To this end, multivariate analyses were conducted with BF% and SMI as independent variables and BMI, BMI at age 20, or the BMI ratio (present to age 20) as dependent variables (Table 4).

BMI was positively associated with SMI (β [95% CI]: 1.6 [1.4, 1.9], *p* < 0.001) and BF% (β [95% CI]: 0.2 [0.1, 0.2], *p* < 0.001); BMI at age 20 was positively associated with age (β [95% CI]: 0.03 [0.006, 0.06], *p* = 0.014), SMI (β [95% CI]: 1.77 [1.06, 2.5], *p* < 0. 001), and body fat percentage (β [95% CI]:1.77 [0.01,0.2], *p* = 0.027); BMI (present to age 20 years) was positively associated with only BF% (β [95% CI]:0.5 [0.05, 0.9], *p* = 0.03). In summary, weight change since the age of 20 years was found to be related to BF%.

#### 3.2.2. SMI and Body Fat Percentage Are Associated with Grip Strength and Lymphocytes, Respectively

Finally, multivariate analyses were performed for items related to nutrition (grip strength, vitamin B_1_ level, cholesterol level, lymphocyte count, and HbA1c level) for Model 1 (age, SMI, and body fat percentage) and Model 2 (age and BMI). Grip strength was positively associated with SMI (β [95% CI]: 4.0 [1.4, 6.6], *p* = 0.003) but not with body fat percentage (*p* = 0.68) (Table 5). There was also no association between grip strength and BMI (β [95% CI]: 0.8 [−0.5, 2.1], *p* = 0.22). The vitamin B_1_ level was not associated with SMI or body fat percentage (β [95% CI]: −0.7 [−4.5, 3.1], *p* = 0.61 and β [95% CI]: 0.1 [−0.3, 0.5] *p* = 0.72, respectively). Vitamin B1 level was also not associated with BMI (β [95% CI]: 0.5 [−1.3, 2.3], *p* = 0.6). Total cholesterol level was associated with age in both Models 1 and 2 (β [95% CI]: 1.2 [0.5, 2.0], *p* = 0.001; and β [95% CI]: 1.3 [0.6, 2.0], *p* < 0.001; respectively) but not with SMI, BF%, or BMI (SMI; β [95% CI]: −2.3 [−24.2, 19.5], *p* = 0.83; body fat percentage: β [95% CI]: −1.0 [−3.4, 1.4], *p* = 0.4; BMI: β [95% CI]: −1.7 [−12.0, 8.6], *p* = 0.74). Lymphocyte count was negatively associated with age (β [95% CI]: −13.9 [−23.3, −4.5], *p* = 0.004; and −15.9 [−25.4, −6.4], *p* = 0.001; respectively) in both Model 1 and Model 2. Furthermore, lymphocyte count was positively associated with BF% (β [95% CI]: 36.2 [6.0, 66.5], *p* = 0.02). Finally, the HbA1c level was not associated with age, SMI, or body fat percentage (age: β [95% CI]: 0.002 [−0.003,0.006], *p* = 0.46; SMI: β [95% CI]:0.05 [−0.08,0.2] *p* = 0.48; body fat%; β [95% CI]:−0.006 [−0.02,0.008], *p* = 0.39, respectively). In Model 2, the HbA1c level was also not associated with BMI or age (β [95% CI]: 0.002 [−0.002, 0.007], *p* = 0.27 and β [95% CI]: 0.002 [−0.06, 0.06], *p* = 0.95, respectively). Thus, grip strength was positively associated with SMI, and lymphocyte count was positively associated with BF%.

## 4. Discussion

In the present study, we aimed to clarify the associations between SMI or BF% and several nutritional markers in underweight women. We determined whether SMI and BF% were associated with markers reflecting nutritional status in women (BMI < 17.5) who were referred to an outpatient nutritional evaluation clinic for checkups. Among the age groups, the frequency of vitamin B1 deficiency was particularly high (22%) in the 40–65-year age group. Cholesterol levels increased with age, whereas lymphocyte count decreased, with a positive CONUT score indicating that the degree of undernutrition increased to 61% at 40 years of age. When the data were divided into SMI quartiles, the grip strength and BMI ratio (present-to-age 20 ratio) increased with increasing SMI, whereas BF% decreased. In the quartile group for BF%, as body fat increased, BMI and the BMI ratio (present to age 20 ratio) increased, and SMI decreased in the opposite direction. According to the multivariate analysis, BMI was positively associated with SMI and BF%, whereas the BMI ratio (present-to-age 20 ratio) was positively associated with BF% but not with SMI. Grip strength was positively associated with SMI, and lymphocyte count was positively associated with BF%. These results indicate that SMI reflects grip strength and that BF% reflects weight change and lymphocyte count from the age of 20 years. These results suggest that it is important to evaluate SMI and BF%, or their respective components (grip strength and triceps subcutaneous fat thickness), in addition to simply measuring body weight when following patients with low nutritional status.

Vitamin B1 deficiency was most common among those aged 40–65 years, as in our previous study [4]. Folic acid deficiency was also most common at 20 years, although the difference was not significant. The levels of vitamin B_12_ and vitamin D were high in all age groups. These results were similar to those of a previous study [4]. Our previous studies revealed that dietary diversity, which is related to nutrient intake and is an indicator of dietary quality, improved with increasing age [4,32]. However, the relatively low rate of fish intake may be responsible for the high frequency of vitamin B_12_ and D deficiency [4,32]. Because folic acid and vitamin B12 are associated with fetal malformations such as neural tube defects [33,34], it is necessary to promote folic acid and vitamin B_12_ intake, especially at younger ages. We have been conducting cooking classes at workplaces to improve food diversity; however, increased diversity with increasing age implies significant socioeconomic influences, such as marriage and eating with a family. The addition of vitamin D to dairy beverages (milk-based drinks) is also available in Japan [35], which may be effective since it provides calcium, vitamin D, and easily absorbed protein [36].

First, SMI and BF% were positively associated with BMI, but a nutritional marker specifically associated with SMI was muscle strength. There was a positive association between grip strength and BF%. Many studies have revealed an association between grip strength and SMI in undernourished patients, although the target patients differ [12,13,14,37,38,39,40,41]. In fact, grip strength is used to diagnose sarcopenia in smaller hospitals, whereas the bioimpedance assay (BIA) or dual X-ray absorptiometry (DXA) method is used to determine SMI in larger hospitals [28]. The patients included in this study were a group of patients with few complications of chronic inflammation, and identifying patients with large fluctuations in SMI from the age of 20 years was difficult. However, since SMI is expected to decrease significantly in a population of undernourished patients complicated by malignant and chronic diseases, the measurement of not only BMI but also SMI and body fat percentage using a body composition analyzer over time is considered important for the evaluation of muscle mass.

Next, one of the nutritional markers specifically related to BF% is the BMI ratio (present-to-age 20 ratio). There was a positive association between the BMI ratio (present-to-age 20 ratio) and BF%. Body weight was lower than that at the age of 20 years (98.8 (5.6) in the 20 s, 98.5 (8.1) in the 30 s, and 96.2 (8.7) in the 40 s). During starvation without underlying disease, muscle loss is very mild, and a decrease in body fat mass occurs, with free fatty acids and ketones used as energy [37]. Therefore, changes in adipose tissue weight, not muscle loss, are attributed to weight changes. On the basis of the above findings, weight changes from 20 to 29 years were thought to be due to changes in fat weight rather than muscle weight.

Finally, one of the nutritional markers specifically related to BF% is lymphocyte count. This study revealed a positive correlation between lymphocyte count and BF%. Lymphocyte count, along with albumin and cholesterol levels, are among the nutritional indicators employed in the CONUT score and are known to decrease with low nutritional status [20,42]. In Japanese women, low body weight is reported to be a risk factor for low lymphocyte counts [4,43]. In adults, approximately 20% to 40% of white blood cells in the body are lymphocytes. These cells help protect the body from infection. If individuals have low numbers of lymphocytes (lymphopenia), they are at increased risk of infection [44]. In nutritional science, a low lymphocyte count is considered to indicate a decrease in immune function [44]. This study is the first to show that lower lymphocyte counts with low nutritional status reflect lower fat weight rather than BMI in Japanese women. Obesity is linked to increased lymphocyte counts, which can contribute to inflammation; furthermore, lymphocyte counts have been reported to be correlated with insulin resistance (homeostatic model assessment of insulin resistance (HOMA-IR)) and BMI [45]. In the adipose tissue of obese patients, lymphocyte infiltration modulates macrophage function, and the number of lymphocytes is correlated with insulin resistance (HOMA-IR) and BMI [46]. In obese patients, lymphocyte infiltration in adipose tissue modulates macrophage function and is implicated in adipose tissue inflammation [46]. A low amount of adipose tissue and therefore a low lymphocyte count can also indicate low inflammation. However, sufficient protein and nutrients are necessary to produce lymphocytes, as protein–energy malnutrition leads to reduced production of lymphocytes [47,48]. Zinc is positively associated with lymphocyte counts [49]. In the future, the possibility of protein–energy wasting (PEW) and zinc deficiency may need to be considered in undernourished patients with a low body fat percentage.

There are several limitations in this study. One limitation of this study is that it is a cross-sectional, observational, exploratory study. Therefore, a valid sample size was not calculated. Second, it should be noted that this study was conducted in a relatively medically literate population. Notably, even in a population with high health literacy, such as nurses and pharmacists, the proportion of underweight women is high, especially in the 20–29-year age group. The fact that the proportion of underweight women increases with age may mean that young women, literate or illiterate, have a poor dietary environment. Future analyses may need to include economic aspects, sleeping hours, and working hours. Second, there was a limited association with biochemical nutritional markers. For example, vitamin D is stored in adipose tissue, so we hypothesized that there might be an association between 25OHD and adipose tissue weight. However, since vitamin D stored in adipose tissue is vitamin D_3_ and 25OHD is present mostly in blood [50], no association was likely. Vitamin B_1_ was not associated with fat or muscle weight because vitamin B_1_ is not readily stored, while folic acid and vitamin B_12_ are present in the liver [51]. HbA1c was also not associated with BF% or SMI because HbA1c was nearly normal. Unsurprisingly, cholesterol is also unrelated to fat and muscle weight, since the liver is the main regulator of cholesterol. Therefore, there was little association between fat weight or muscle and biochemical markers.

Third, the present study was limited to underweight young and middle-aged women. To generalize the results to men, analysis involving men is also needed. Fourth, because we used a home-use Inbody device, it has limitations as a method for measuring muscle mass and adiposity. The reason for using a home-use machine is that the results of this study can applied to regular clinics that are not expensive. Compared with the home-use Inbody device, professional BIA measuring devices used in hospitals offer advantages such as higher frequency and greater water content; however, in reality, in cases with increased body fluid volume, the phase angle is often used to evaluate muscle mass, even with professional BIA methods [52]. There are other methods of assessing body composition, such as the DXA method, but this method also calculates the water content at 73%, meaning that edema and dehydration can cause measurement errors [28,52]. Although the subjects in this study did not have edema, and therefore, no problems arose, it is thought that the Inbody device for home use is less accurate than the Inbody device for professional use. Nevertheless, all methods have limitations related to changes in body fluid volume.

## 5. Conclusions

We examined the associations between body size and nutritional indices in underweight women. We found that SMI and BF% play different roles in grip strength and lymphocyte counts, respectively. These findings suggest that SMI and BF% are nutritional markers that are independent of BMI. This study also revealed a link between lymphocyte counts and BF%. Further investigations are needed to clarify the relationship between lymphopenia and lower BF%. Future investigations involving minerals such as zinc are needed to clarify this mechanism. Focusing medical care not only on BMI but also on SMI and BF% may lead to a comprehensive nutritional assessment that includes muscle strength and immune function.

## Figures and Tables

**Table 1:** Background of the participants in this study.

		Age				SMI					BF%				
	Total (n = 102)	20–29 yo (n = 64)	30–39 yo (n = 20)	40–65 yo (n = 18)	*p*	Q1 (n = 26)	Q2 (n = 25)	Q3 (n = 25)	Q4 (n = 26)	*p*	Q1 (n = 26)	Q2 (n = 26)	Q3 (n = 24)	Q4 (n = 26)	*p*
Age (years)	30.9 (10.2)	24.6 (1.7)	33.4 (3.0)	50.3 (6.8)	<0.001	28.6 (9.9)	31.0 (9.2)	32.3 (10.6)	31.8 (11.3)	0.58	33.0 (10.3)	31.8 (12.7)	28.0 (5.6)	30.5 (10.7)	0.36
BMI (kg/m^2^)	17.0 (0.7)	17.0 (0.7)	16.8 (0.7)	17.2 (0.6)	0.32	16.6 (0.8)	16.8 (0.8)	17.3 (0.6)	17.3 (0.4)	<0.001	16.7 (0.8)	17.0 (0.6)	17.0 (0.8)	17.3 (0.6)	0.04
BMI at 20 years (kg/m^2^)	17.4 (1.4)	17.3 (1.2)	17.2 (1.7)	18.1 (1.6)	0.09	16.5 (1.0)	17.2 (1.1)	17.7 (0.1)	18.1 (7.8)	<0.001	17.6 (1.7)	17.5 (1.4)	17.1 (1.4)	17.3 (1.0)	0.5
BMI ratio (%)	98.3 (6.7)	98.8 (5.6)	98.5 (8.1)	96.2 (8.7)	0.36	100.6 (5.1)	98.4 (6.2)	98.0 (7.1)	96.3 (7.8)	0.15	95.3 (7.3)	97.8 (6.4)	100.0 (5.6)	100.4 (6.5)	0.032
SMI (kg/m^2^)	7.1 (0.4)	7.0 (0.5)	7.1 (0.4)	7.2 (0.4)	0.2	6.5 (0.2)	7.0 (0.07)	7.2 (0.1)	7.6 (0.2)	<0.001	7.4 (0.4)	7.2 (0.4)	7.0 (0.3)	6.7 (3.2)	<0.001
BF%	22.0 (4.0)	22.6 (4.1)	20.8 (2.6)	21.2 (4.5)	0.13	25.2 (4.1)	22.4 (3.9)	21.6 (2.6)	18.9 (2.3)	<0.001	17.5 (1.4)	20.4 (0.5)	22.7 (1.2)	27.5 (2.2)	<0.001
Grip strength (kg)	22.5 (4.8)	22.3 (5.0)	21.4 (4.6)	24.1 (3.7)	0.2	20.0 (3.6)	22.0 (4.4)	23.2 (4.1)	24.7 (5.6)	0.03	23.9 (4.6)	23.3 (5.0)	21.2 (4.4)	21.5 (4.7)	0.12

Abbreviations: BMI, body mass index; SMI, skeletal muscle mass index; BF%, body fat percentage.

**Table 2:** Vitamin levels and the frequencies of vitamins in underweight women.

		Age				SMI					BF%				
	Total (n = 102)	20–29 yo (n = 64)	30–39 yo (n = 20)	40–65 yo (n = 18)	*p*	Q1 (n = 26)	Q2 (n = 25)	Q3 (n = 25)	Q4 (n = 26)	*p*	Q1 (n = 26)	Q2 (n = 26)	Q3 (n = 24)	Q4 (n = 26)	*p*
Β_1_ [24–66] (ng/mL)	30.1 (6.6)	30.7 (6.3)	30.0 (7.7)	28.1 (6.1)	0.33	32.0 (8.1)	28.9 (4.8)	29.3 (7.1)	30.0 (5.6)	0.32	31.2 (9.2)	29.2 (4.6)	27.3 (4.1)	32.3 (6.29)	0.032
Normal	94 (92%)	60 (94%)	20 (100%)	14 (78%)	0.03	25 (96%)	23 (92%)	23 (92%)	23 (89%)	0.79	22 (85%)	25 (96%)	21 (87%)	26 (100%)	0.14
Deficiency	8 (8%)	4 (6%)	0 (0%)	4 (22%)		1 (4%)	2 (8%)	2 (8%)	3 (11%)		4 (15%)	1 (4%)	3 (13%)	0 (0%)	
Folate [≥4] (ng/mL)	8.1 (4.8)	7.4 (4.4)	9.7 (5.9)	8.7 (4.3)	0.13	8.0 (4.1)	8.1 (5.8)	8.8 (5.1)	7.3 (4.1)	0.73	8.6 (4.3)	6.8 (3.8)	8.2 (5.8)	8.7 (5.2)	0.47
Normal	90 (88%)	53 (83%)	20 (100%)	17 (94%)	0.08	23 (89%)	21 (84%)	24 (96%)	22 (85%)	0.53	23 (88%)	23 (88%)	22 (92%)	22 (85%)	0.9
Deficiency	12 (12%)	11 (17%)	0 (0%)	1 (6%)		3 (12%)	4 (16%)	1 (4%)	4 (15%)		3 (12%)	3 (12%)	2 (8%)	4 (15%)	
B_12_ [200–914] (pg/mL)	289.4 (131.0)	271.1 (115.8)	338.1 (148.6)	299.3 (152.4)	0.13	267.8 (99.6)	277.2 (144.7)	323.9 (141.9)	288.7 (133.6)	0.46	294.2 (118.3)	262.3 (94.2)	346.3 (181.3)	258.1 (104.8)	0.066
Normal	77 (76%)	47 (73%)	16 (80%)	14 (78%)	0.81	20 (77%)	16 (64%)	21 (84%)	20 (77%)	0.42	19 (73%)	21 (81%)	20 (83%)	17 (65%)	0.44
Deficiency	25 (24%)	17 (27%)	4 (20%)	4 (22%)		6 (23%)	9 (36%)	4 (16%)	6 (23%)		7 (27%)	5 (19%)	4 (17%)	9 (35%)	
25OHD [≥30] (ng/mL)	11.2 (5.0)	11.0 (4.7)	12.1 (5.3)	11.0 (6.1)	0.7	10.4 (3.9)	10.6 (4.9)	13.2 (6.0)	10.8 (4.9)	0.16	12.7 (6.5)	10.6 (4.5)	10.5 (4.0)	11.2 (4.8)	0.39
Normal	6 (6%)	3 (5%)	1 (5%)	2 (11%)	0.58	0 (0%)	1 (4%)	3 (12%)	2 (8%)	0.30	23 (88%)	24 (92%)	24 (100%)	25 (96%)	0.34
Deficiency	96 (94%)	61 (95%)	19 (95%)	16 (89%)		26 (100%)	24 (96%)	22 (88%)	24 (92%)		3 (12%)	2 (8%)	0	1 (4%)	

The normal range was represented as [XX]. The data are presented as the means ± SDs. Plasma vitamin B_1_ deficiency, vitamin B_12_ deficiency, plasma folate deficiency, and vitamin D deficiency were defined as plasma vitamin B_1_ levels < 24 ng/mL, plasma vitamin B_12_ levels < 200 pg/mL, plasma folate levels < 4 ng/mL, and plasma 25(OH)D levels < 20 ng/mL, respectively.

**Table 3:** Nutritional markers in underweight women.

		Age				SMI					BF%				
	Total (n = 102)	20–29 yo (n = 64)	30–39 yo (n = 20)	40–65 yo (n = 18)	*p*	Q1 (n = 26)	Q2 (n = 25)	Q3 (n = 25)	Q4 (n = 26)	*p*	Q1 (n = 26)	Q2 (n = 26)	Q3 (n = 24)	Q4 (n = 26)	*p*
Total cholesterol [120–219] (mg/dL)	179.2 (39.5)	174.8 (26.7)	166.1 (16.5)	209.4 (71.2)	<0.001	183.9 (27.6)	169.2 (19.4)	180.6 (26.2)	182.8 (66.1)	0.53	186.3 (62.3)	181.2 (33.8)	170.8 (19.4)	177.9 (28.2)	0.57
Albumin (g/dL)	4.5 (0.3)	4.5 (0.3)	4.5 (0.2)	4.4 (0.3)	0.4	4.5 (0.2)	4.5 (0.3)	4.5 (0.4)	4.5 (0.3)	0.97	4.5 (0.3)	4.4 (0.3)	4.5 (0.3)	4.5 (0.3)	0.35
Lymphocyte [1000–4800] (/μL)	1760.0 (510.2)	1859.3 (493.8)	1739.0 (503.4)	1429.6 (454.1)	0.006	1846.1 (530.5)	1787.7 (485.0)	1683.6 (567.8)	1720.2 (467.8)	0.68	1620.2 (385.8)	1732.1 (522.9)	1751.3 (320.4)	1935.3 (694.0)	0.16
CONUT score															
Normal (0–1)	62 (61%)	45 (70%)	10 (50%)	7 (39%)	0.03	19 (73%)	16 (64%)	14 (56%)	13 (50%)	0.35	12 (46%)	16 (62%)	18 (75%)	16 (62%)	0.22
Mild (2–4)	40 (39%)	19 (30%)	10( 50%)	11 (61%)		7 (27%)	9 (36%)	11 (44%)	13 (50%)		14 (54%)	10 (38%)	6 (25%)	10 (38%)	
Prealbumin [22–40] (mg/dL)	23.6 (4.1)	23.4 (4.3)	23.7 (3.6)	24.0 (4.0)	0.84	23.3 (4.0)	23.2 (4.4)	24.5 (4.2)	23.3 (3.8)	0.65	23.2 (4.0)	24.0 (4.7)	22.1 (3.9)	24.8 (3.4)	0.12
HbA1c [4.6–6.2] (%)	5.4 (0.2)	5.4 (0.2)	5.5 (0.2)	5.5 (0.2)	0.51	5.4 (0.3)	5.4 (0.2)	5.5 (0.2)	5.5 (0.2)	0.049	5.5 (0.2)	5.5 (0.2)	5.5 (0.2)	5.4 (0.2)	0.19
Hb [11.4–14.6] (g/dL)	13.0 (1.0)	13.0 (0.8)	12.4 (1.5)	13.4 (0.9)	0.006	13.0 (0.9)	12.7 (1.4)	12.9 (0.9)	13.1 (0.8)	0.65	13.1 (1.1)	12.7 (0.9)	12.8 (1.2)	13.3 (0.9)	0.13
Normal	91 (89%)	58 (91%)	15 (75%)	18 (100%)	0.039	22 (85%)	22 (88%)	22 (88%)	25 (96%)	0.58	25 (96%)	21 (81%)	21 (87%)	24 (92%)	0.31
Anemia	11 (11%)	6 (9%)	5 (25%)	0 (0%)		4 (15%)	3 (12%)	3 (12%)	1 (4%)		1 (4%)	5 (19%)	3 (13%)	2 (8%)	

The normal range was represented as [XX]. The data are presented as the means ± SDs. Plasma vitamin B_1_ deficiency, vitamin B_12_ deficiency, plasma folate deficiency, and vitamin D deficiency were defined as plasma vitamin B_1_ levels < 24 ng/mL, plasma vitamin B_12_ levels < 200 pg/mL, plasma folate levels < 4 ng/mL, and plasma 25(OH)D levels < 20 ng/mL, respectively.

**Table 4:** Multivariate regression analysis of the associations between SMI, BF%, and BMI.

Independent Variable	Dependent Variable	β (95% CI)	*p*
BMI (kg/m^2^)	Age	0.007 [−0.001, 0.02]	0.099
	SMI (kg/m^2^)	1.6 [1.4, 1.9]	<0.001
	BF%	0.2 [0.1, 0.2]	<0.001
BMI (kg/m^2^) at 20 yo	Age	0.03 [0.006, 0.06]	0.014
	SMI (kg/m^2^)	1.77 [1.06, 2.5]	<0.001
	BF%	1.77 [0.01, 0.2]	0.027
BMI ratio (vs. 20 yo)	Age	−0.1 [−0.2, 0.01]	0.08
	SMI (kg/m^2^)	−0.4 [−4.2, 3.6]	0.83
	BF%	0.5 [0.05, 0.9]	0.03

Multivariate analysis was performed with BMI, weight at age 20, and the ratio of current weight to age 20 weight as dependent variables, and SMI, BF%, and age as independent variables. Abbreviations: BMI, body mass index; SMI, skeletal muscle mass index; BF%, body fat percentage.

**Table 5:** Multivariate regression analysis of the associations between SMI or BF% and nutritional markers.

		MODEL 1		MODEL 2	
Independent Variable	Dependent Variable	β (95% CI)	*p*	β (95% CI)	*p*
Grip strength (kg)	Age	0.05 [−0.04, 0.1]	0.24	0.07 [−0.02, 0.2]	0.13
	SMI (kg/m^2^)	4.0 [1.4, 6.6]	0.003		
	BF%	0.06 [−0.2, 0.3]	0.68		
	BMI			0.8 [−0.5, 2.1]	0.22
B_1_ (ng/mL)	Age	−0.07 [−0.2, 0.06]	0.32	−0.08 [−0.2, 0.05]	0.22
	SMI (kg/m^2^)	−0.7 [−4.5, 3.1]	0.61		
	BF%	0.1 [−0.3, 0.5]	0.72		
	BMI			0.5 [−1.3, 2.3]	0.6
Total cholesterol (mg/dL)	Age	1.2 [0.5, 2.0]	0.001	1.3 [0.6, 2.0]	<0.001
	SMI (kg/m^2^)	−2.3 [−24.2, 19.5]	0.83		
	BF%	−1.0 [−3.4, 1.4]	0.4		
	BMI			−1.7 [−12.0, 8.6]	0.74
Lymphocyte (/μL)	Age	−13.9 [−23.3, −4.5]	0.004	−15.9[−25.4, −6.4]	0.001
	SMI (kg/m^2^)	118.5 [−158.7, 395.8]	0.4		
	BF%	36.2 [6.0, 66.5]	0.02		
	BMI			90.7 [−42.5, 224.0]	0.18
HbA1c (%)	Age	0.002 [−0.003, 0.006]	0.46	0.002 [−0.002, 0.007]	0.27
	SMI (kg/m^2^)	0.05 [−0.08, 0.2]	0.48		
	BF%	−0.006 [−0.02, 0.008]	0.39		
	BMI			0.002 [−0.06,0.06]	0.95

Multivariate analyses were conducted with grip strength, vitamin B_1_ level, total cholesterol level, lymphocyte count, and HbA1c level as dependent variables, and SMI, BF%, and age (Model 1) or BMI and age (Model 2) as independent variables. Abbreviations: BMI, body mass index; SMI, skeletal muscle mass index; BF%, body fat percentage.

## Data Availability

Some or all datasets generated and/or analyzed during the current study are not publicly available but are available from the corresponding author upon reasonable request.

## Abbreviations

The following abbreviations are used in this manuscript:

SMI	Skeletal muscle mass index
BF%	Body fat percentage
CONUT	CONtrolling NUTritional status
